# Facial nerve regeneration using silicone conduits filled with ammonia-functionalized graphene oxide and frankincense-embedded hydrogel

**DOI:** 10.1186/s41232-021-00162-x

**Published:** 2021-04-26

**Authors:** Sepehr Aghajanian, Aliasghar Taghi Doulabi, Masoume Akhbari, Alireza Shams

**Affiliations:** 1 Student Research Committee, Alborz University of Medical Sciences, Karaj, Iran; 2 Alborz University of Medical Sciences, Karaj, Iran; 3 Department of Anatomy, School of Medicine, Alborz University of Medical Sciences, Karaj, Iran

**Keywords:** Nerve repair, Facial nerve, Frankincense, Graphene oxide, Regeneration

## Abstract

**Background:**

Silicone tube (ST) conduits have been accepted as a therapeutic alternative to direct nerve suturing in the treatment of nerve injuries; however, the search for optimal adjuncts to maximize the outcomes is still ongoing. Frankincense (Fr) and graphene oxide (GO) have both been cited as neuroregenerative compounds in the literature. This study assesses the efficacy of these materials using a ST conduit in a rat facial nerve motor neuron axotomy model, distal to the stylomastoid foramen.

**Methods:**

Ammonia-functionalized graphene oxide (NH2-GO) and/or Fr extract were embedded in a collagen-chitosan hydrogel and were injected inside a ST. The ST was inserted in the gap between the axotomized nerve stumps. Return of function in eye closure, blinking reflex, and vibrissae movements were assessed and compared to control groups through 30 days following axotomy. To assess the histological properties of regenerated nerves, biopsies were harvested distal to the axotomy site and were visualized through light and fluorescence microscopy using LFB and anti-MBP marker, respectively.

**Results:**

There was no significant difference in behavioral test results between groups. Histological analysis of the nerve sections revealed increased number of regenerating axons and mean axon diameter in NH2-GO group and decreased myelin surface area in Fr group. Using both NH2-GO and Fr resulted in increased number of regenerated axons and myelin thickness compared to the hydrogel group.

**Conclusions:**

The findings suggest a synergistic effect of the substances above in axon regrowth, notably in myelin regeneration, where Fr supposedly decreases myelin synthesis.

## Introduction

Facial nerve (CN VII) paralysis, while not being life-threatening, can be drastically disabling to the patients and profoundly impacting the psychosocial aspects of life [1]. Despite the considerable advances of recent years and new microsurgical techniques proposed in the literature, facial nerve reconstruction remains a challenge for reconstructive surgery [2]. While facial nerve possesses the regenerative capacity of other peripheral nerves [3], the results of established treatments have been less than satisfactory [4]. Numerous reconstructive surgeries are available to repair nerve transection, including direct anastomosis of the distal and proximal parts of severed nerve, end-to-end and end-to-side coaptation with or without interposition nerve grafting [5, 6], and tubulization with biological or synthetic conduits [7–9]. Whereas autografts remain the gold standard for nerve repair [10], comparable results can occasionally be obtained by employing both biological and nonbiological conduits [7]. Interestingly, tissue-engineered conduits can sometimes outperform traditional neurorrhaphy techniques [7, 11].

Nevertheless, artificial nerve conduits have their own shortcomings, and the search for finding the optimal biomaterials for these conduits carries on. Thus, this study was conducted to assess the efficacy of novel adjuncts in hydrogels in regeneration and regrowth of motor neuron axons in a facial nerve axotomy model. Silicone rubber was chosen as the material of choice for the conduits in our model due to its accessibility, compatibility with short nerve gaps and along with a non-crosslinked collagen-chitosan-based hydrogel, the ability to be used as a template for a rudimentary assessment of the regenerative properties of the substances of interest in this study.

Graphene oxide (GO), as a derivative of graphene, is synthesized through extensive oxidation of graphite and consequent exfoliation to single layers. Carboxylate groups in GO provide colloidal stability and resistance to precipitation in aqueous solution compared to pure graphene [12]. Therefore, GO can be more likely utilized in water-based hydrogels without the need of non-polar solvents.

GO-containing scaffolds induce neural stem cell and embryonic stem cell differentiation into neurons and promote neuronal growth in vitro [13, 14]. Incorporation of GO inside hydrogels has improved their physico-chemical properties in a multitude of studies in a variety of tissues [15, 16] and there is also evidence that both graphene and GO increase neurite outgrowth in vitro [14, 17, 18]. These substances may facilitate axonal sprouting through upregulation of focal adhesion kinase and mitogen-activated protein kinase (MAPK or ERK) pathways [10, 19] leading to increased Hsp27 phosphorylation in neuronal cells [20]. GO scaffolds have also been used to improve the outcome of nerve repair in vivo [10, 21]. While there is conflicting information about the toxicity of graphene oxide, the majority of in vitro studies hint at possible in vitro reactive oxygen species (ROS) generation and oxidative stress at high concentrations of GO [12]. Moreover, studies have shown that surface modification and functionalization of GO, in which the carboxyl groups in GO structure are partially replaced by other functional group, alter its toxicity and in vivo distribution following implantation [22]. Furthermore, ammonia-functionalized GO (NH2-GO) was also demonstrated to increase neurite sprouting in hippocampal neurons in vitro compared to chemical functionalization with other groups [17]. Therefore, surface modification of GO has a greater potential to be used as biodegradable implantation materials for neural regeneration. Thus, we opted to use an ammonia-functionalized GO instead of the pure or other functionalized GO compounds.

Frankincense (Fr) also called olibanum is an oleo-gum-resin obtained from the trees in genus Boswellia. The oleo-gum-resin of *B. serrata* has been used in controlling headaches and inflammatory conditions such as arthritis and colitis, and more recently introduced as an anticancer agent [23–25]. Administering purified compounds from Boswellia extracts showed contradicting and sometimes inferior results compared to crude extracts in the treatment of inflammation [25]. For example, Fr essential oil commonly acquired from *B. carterii* is demonstrated to reduce corticosteroid hormones, whereas its major components, limonene and α-pinene, elevated levels of corticosterone [26]. Studies also confirmed the superior activity of the oleo-gum-resin compared to purified boswellic acids [25, 27, 28].

There is compelling evidence that Boswellia resin has neuroprotective and anti-inflammatory properties. Currently identified mechanisms promoting anti-inflammatory effects of boswellic acids such as acetyl-β-boswellic acid and 3-acetyl-11-keto-β-boswellic acid (AKBA) are inhibition of leukotriene synthesis through non-competitive inhibition of 5-lipoxygenase and reversible inhibition of COX-1 and COX-2 enzymes, reduction of pro-inflammatory cytokines (TNF, IL-1, IL-2, IL-6, and IFN-γ), inhibition of NF-κB signaling activation, complement system inhibition, and blood-brain barrier regulation via preservation of intercellular junctions [24, 25, 29–31]. While anti-inflammatory properties of the resin have indirect neuroprotective effects; other mechanisms have been mentioned in which Fr either mitigate damage or promote regeneration in neurons. Beta-boswellic acid is shown to interfere with Alzheimer’s disease pathway, probably through hydrophobic binding with *tau* protein [32]. Fr is also demonstrated to increase outgrowth and branching of neurites in hippocampal neurons [33]. Another triterpenoid compound, 11-keto-β-boswellic acid (KBA), reduces oxidative stress by upregulating nuclear factor erythroid-2-related factor 2 (NRF2)/heme oxygenase-1 pathway in astrocytes [34]. Furthermore, studies show that ERK phosphorylation is increased in Schwann cells, which contributes to peripheral nerve regeneration via Schwann cell proliferation [35]. AKBA is also observed to reduce glutamate toxicity by reducing levels of ROS and inhibiting apoptotic activity [36]; the same mechanisms also contribute to reduced damaged to nigrostriatal dopaminergic neurons in Parkinson’s disease model in rats [23]. Oral administration of Fr extract is also demonstrated to reduce MRI-identified lesions in long-term usage in patients with multiple sclerosis [37] and increase BDNF expression in adult rats [38].

In this study, we tried to verify whether the anti-inflammatory properties of Fr and its potentiation of Schwann cells, which are vital components in nerve regeneration and myelination [39], amplify the neuroregeneration of ammonia-functionalized GO while attenuating the cellular damage induced by oxidative stress due to GO and nerve injury [12, 40].

## Materials and methods

### Ammonification of graphene oxide

Graphene oxide was functionalized and partially reduced via the one-pot synthesis method provided by Lai et al. [41] as below.

One hundred milligrams of GO was dissolved in 40 ml of ethylene glycol through ultrasonication. GO procured locally was synthesized based on Hummers’ procedure [42]. One milliliter of NH4OH was added before transferring the solution to a Teflon-lined autoclave for solvothermal reaction at 180 °C for 10 h. The precipitate was then gathered and dried at 60 °C for 24 h. The resulting functionalized GO (NH2-GO) was dissolved in distilled water and stored for further use in 1 mg/ml concentration. The detailed structure and electrochemical properties of the final substance are characterized by X-ray photoelectron spectroscopy, proton nuclear magnetic resonance, and Fourier-transform infrared spectroscopy in the original study [41].

### Preparation of frankincense water extract

*Boswellia serrata* oleo-gum-resin was purchased from a trusted herbal pharmacy store and ground into powder. One hundred milligrams of powder was added to 300 ml of distilled water and stored for 24 h in 4°C before being heated in laboratory water bath. The solution was filtered and stored in 4°C for later use.

### Hydrogel composition

To prepare the hydrogel, chitosan (Sigma-Aldrich, CAS Number 9012-76-4) and collagen (100 mg/ml) extracted from rat tail as previously reported [43] were obtained.

Thirty-four grams of chitosan was added to 500 μl of PBS (Sigma-Aldrich) and stirred with a magnet mixer for 10 min. Two hundred microliters of collagen solution was added to prepare the basic hydrogel. For experimental groups, 200 microliters of GO-NH2 and/or 500 microliters of Fr extract were used along with collagen and chitosan. Finally, the volume of hydrogel was raised to 2 ml with PBS and titrated to biological pH with hydrochloric acid (pH ≈7.32). The hydrogel was sterilized under UV-C light for 30 min prior to administration.

### Intervention and evaluation

**Fig. 1:**
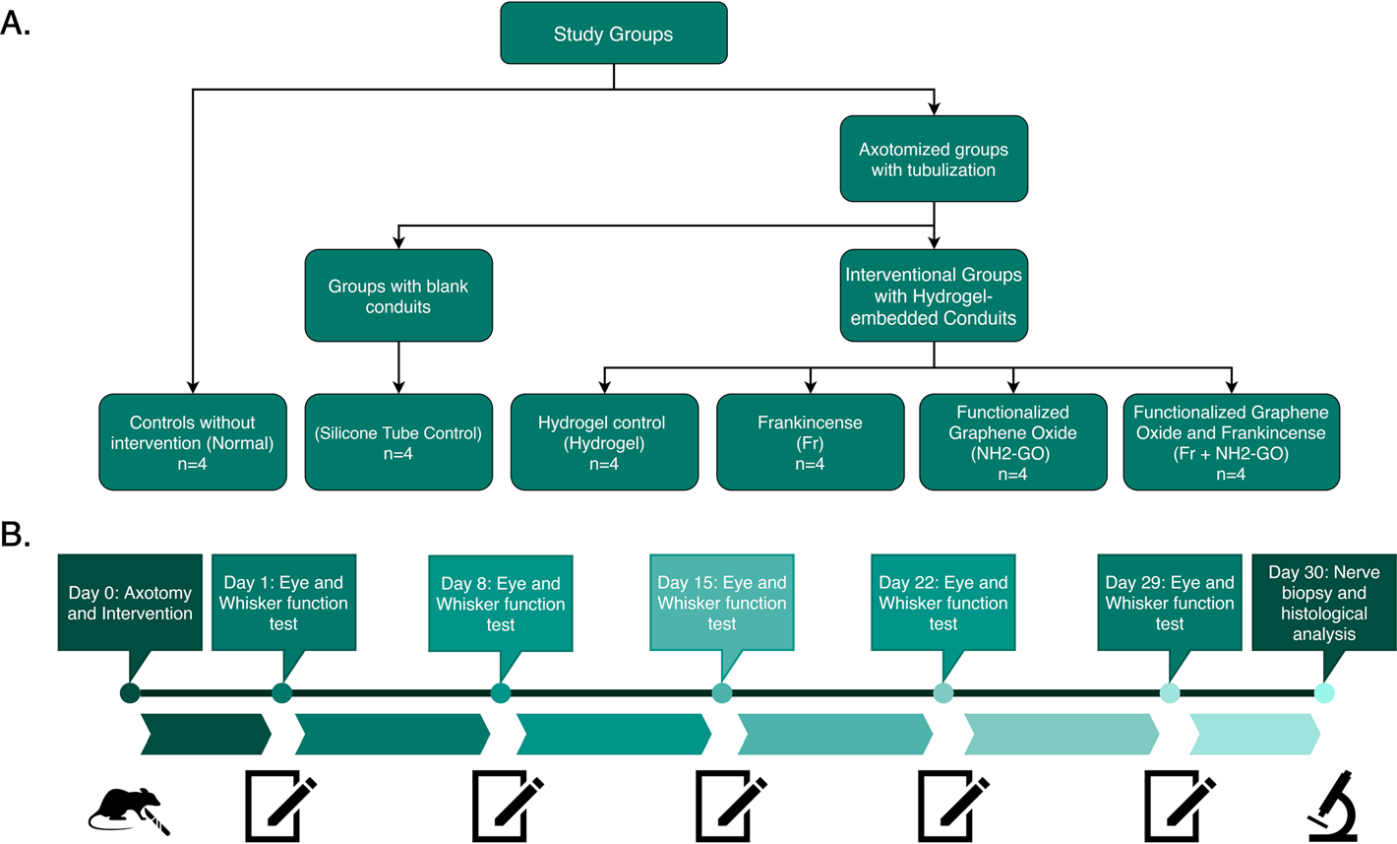
Study groups (**a**) and timeline (**b**). Nerve biopsies were obtained and fixed with paraformaldehyde 30 days following axotomy and tubulization

Twenty-four male Wistar rats (10 weeks old) weighing 300 ± 50 g used in this study were obtained from Pasteur’s Institute (Tehran, Iran). All procedures used in this study were in accordance with Ethics committee guidelines for Animal Experiments at Alborz University of Medical Sciences (approval no. 1398.067). Animals were randomly divided into two control and four experimental groups (*n*=4). The *normal* group had no intervention and was kept with other groups. All other groups underwent bilateral facial nerve axotomy and left sided nerve tubulization with a silicone tube conduit. The silicone tube was left blank for the *silicone tube control* group and was filled with the basic hydrogel for the *hydrogel* group. NH2-GO and Fr extract were added to the hydrogel for *Fr* and *NH2-GO* groups, respectively. Both substances were added to the hydrogel for *Fr+NH2-GO* group. The study groups and timelines are illustrated in Fig. 1.

The animals were anesthetized with an intraperitoneal injection of ketamine (100 mg/ml, 3 ml) and xylazine (100 mg/ml, 1 ml). The incision was made below the base of ear, inferior to the ear cartilage. The digastric muscle and parotid gland were dissected for easier access to the nerve. In left side of the face, facial nerve was then held in place by threading two 7-0 nylon sutures through the nerve, immediately below epineurium, and in close proximity to prevent springing following axotomy. The main trunk was severed between the threaded sutures, distal to the stylomastoid foramen. Proximal and distal parts of the main trunk were then pulled to a sterile 5 mm blank or filled silicone tube (ID≈1.75mm - OD≈2.35mm) based on designated specifications for each group. A 2 mm gap was left between the stumps to accommodate hydrogel and facilitate intratubular regeneration. The thread ends were tied together to prevent the dislocation of the stumps. In the right side of the face of the experimental groups, axotomy took place to eliminate cross-innervation in whisker pads without further intervention [44]. Fascia and surrounding and anatomical structures were returned to their original location, and the wound was sutured with 5-0 nylon suture. All groups were treated with a 3-ml single dose of gentamicin (40 mg/ml) after the surgery. Animals were housed in cages in groups of two with adjusted ambient temperature, relative humidity (25 °C—50%), and 12 h-12 h dark-light cycles. Food and water were granted ad libitum.

### Eye closure and whisker function test

Animals’ recovery was evaluated in days 1, 8, 15, 22, and 29 after axotomy with a behavioral test based on de Faria et al.’s proposed procedure [45]. To assess eye closure, blinking reflex, and vibrissae movement, each animal is placed in an isolated cage. Eye closure and whisker function were graded with a 5-score scale as stated in Table 1. To incite blinking and triggering reflex, each rat was stimulated with air inflation (20 ml) on the face. To evaluate vibrissae movements, proper excitatory stimulus (hand clapping) was given prior to examination. All tests were conducted by the same observers, repeated multiple times for consistency, and scored compared to normal controls (score 5). The observers were blinded to the experimental groups.

**Table 1:** Facial nerve functional behavioral test observation scale. Adapted from [45]

Vibrissae movement assessment scale			Eye closure and blink reflex observation scale
Score	Movement	Vibrissae position	Movement
1	No movement	Posterior	No movement
2	Light tremor	Posterior	Contraction/no closure
3	Greater tremor	Posterior	50% closure
4	Normal movement	Posterior	75% closure
5	Normal movement	Anterior	Complete closure

### Histological examination and immunostaining

The silicone tubes containing regenerated nerves were harvested from the left side of the face 30 days after the initial intervention and were fixed with a solution containing 4% paraformaldehyde. Afterward, the nerve sections distal to the tube site were dehydrated through a graded ethanol series and were cut into 5-μm thick sections after being embedded in paraffin. The cross-section slides were stained with Luxol fast blue (LFB) to compare the number of regenerated nerves, myelin thickness per nerve fiber, and axon diameter of samples of each group using light microscopy.

To prepare the tissue samples for immunostaining procedures, the paraffin-embedded blocks were sections into 3-μm thickness and transferred to slides. The slides were deparaffinized and rehydrated using a graded ethanol and xylene series. The samples were then rinsed in PBS (3×5 min), underwent antigen retrieval using citrate buffer (incubation at 95 °C for 10 min), blocked for 1 h in PBS with 10% normal goat serum and 0.3% Triton X-100 and incubated at 2-8 °C for 24 h with anti-myelin basic protein (MBP, Abcam #ab62631, 1∶100) antibody. After washing with PBS, the sections were incubated with appropriate secondary antibodies (goat anti-mouse AlexFlour, green) at 37 °C for 90 min. The slides were washed with PBS (3×5min) before and after applying DAPI staining to observe the cell nuclei. The stained sections were assessed under a fluorescent microscope (Olympus BX51, Japan) using the ImageJ software for the intensity of the marker, reported as percentage of surface area of the section with positive illumination. The results of the interventional groups were compared to hydrogel group.

### Statistical analysis

Differences among the data of control and experimental groups for behavioral and histological tests were analyzed by one-way and two-way ANOVA statistical analysis, respectively. Normality assumption and equality of variances was tested and confirmed by the Levene’s test (data not shown). The analysis was carried out using GraphPad Prism 9.0 statistical analysis software (GraphPad Software, Inc., La Jolla, CA, USA). *p*<0.05 in each comparison was considered to indicate significance.

## Results

**Fig. 2:**
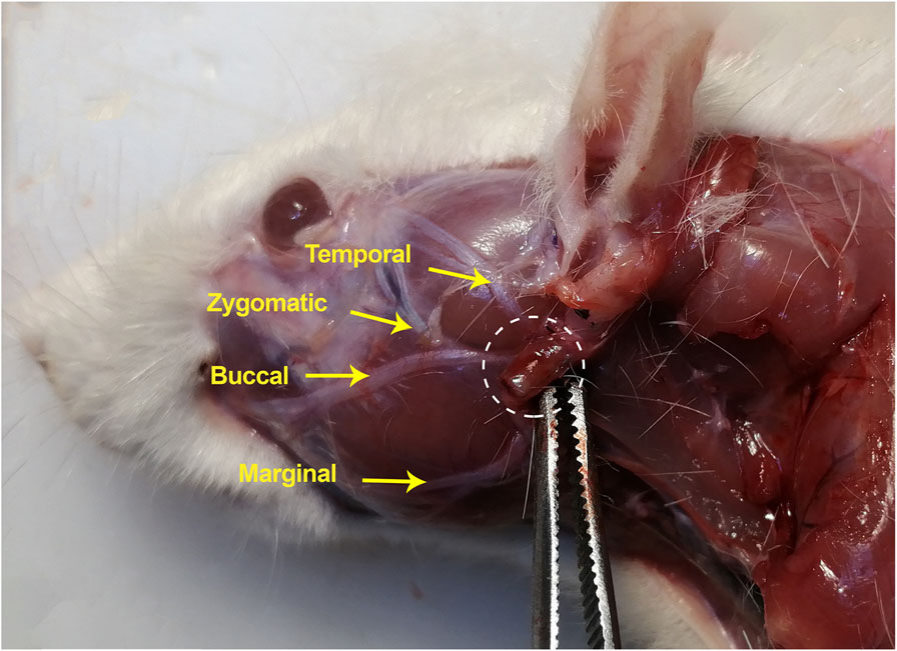
Location of the silicone tube insertion in experimental groups, silicone tube is retracted rostrally and is denoted by dashed lines. The overlying anatomy was removed for a clear representation. Facial nerve branches are demonstrated by the arrows

All animals were euthanized by CO2 inhalation in isolated cages 30 days after the axotomy. The silicone tubes were embedded in the surrounding tissue (Fig. 2). No swelling or deformation of the silicone tubes and no dislocation of the nerve stumps was observed. Nerve formation and regrowth inside the silicone tube was spotted in gross examination of all extracted conduits.

**Fig. 3:**
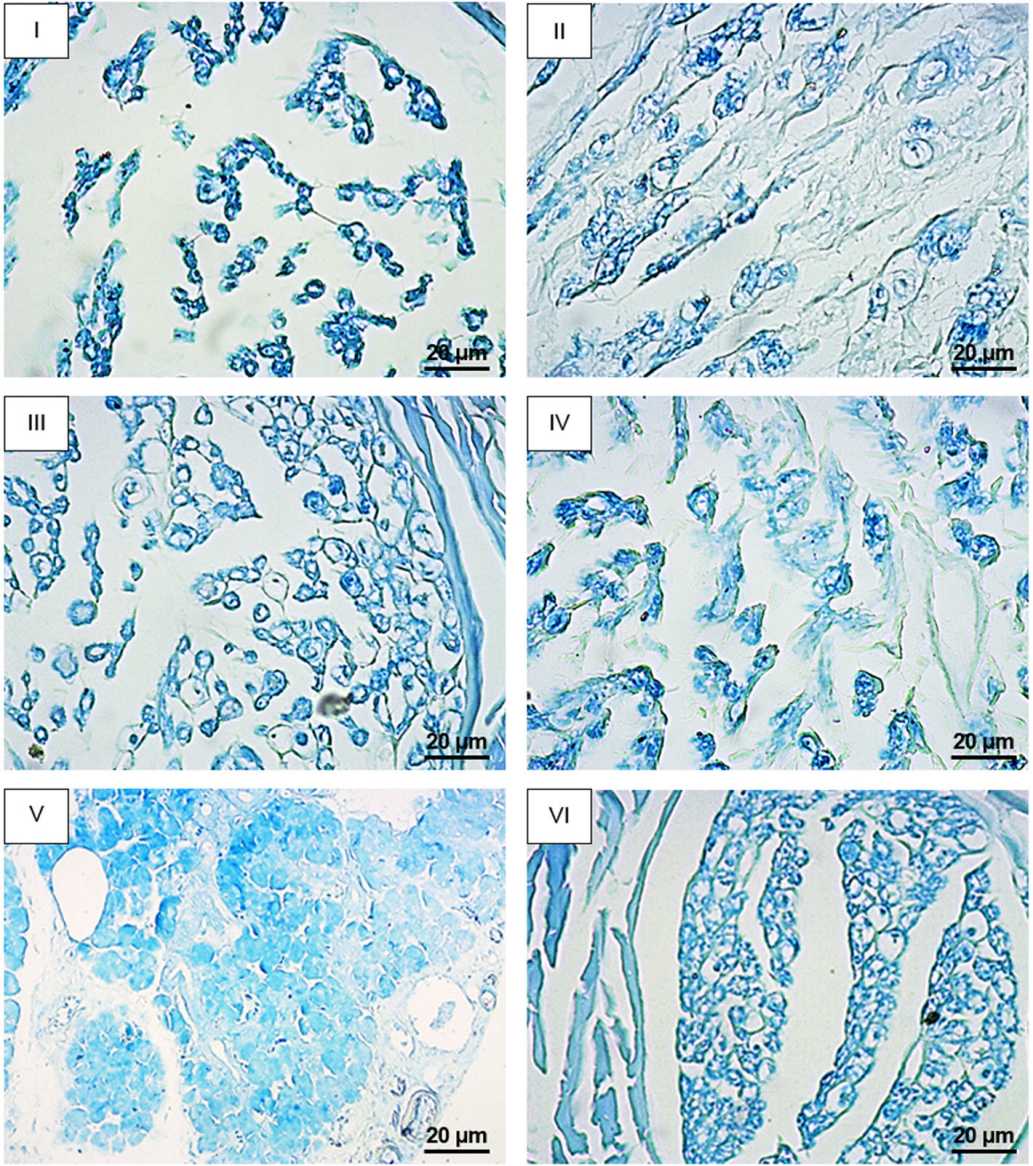
Luxol fast blue stained nerve cross-sections in different groups. I: silicone tube control; II: hydrogel; III: Fr; IV: NH2-GO; V: Fr + NH2-GO; VI: normal. Reduced axonal regrowth is characterized by increased intercellular space in control groups

**Fig. 4:**
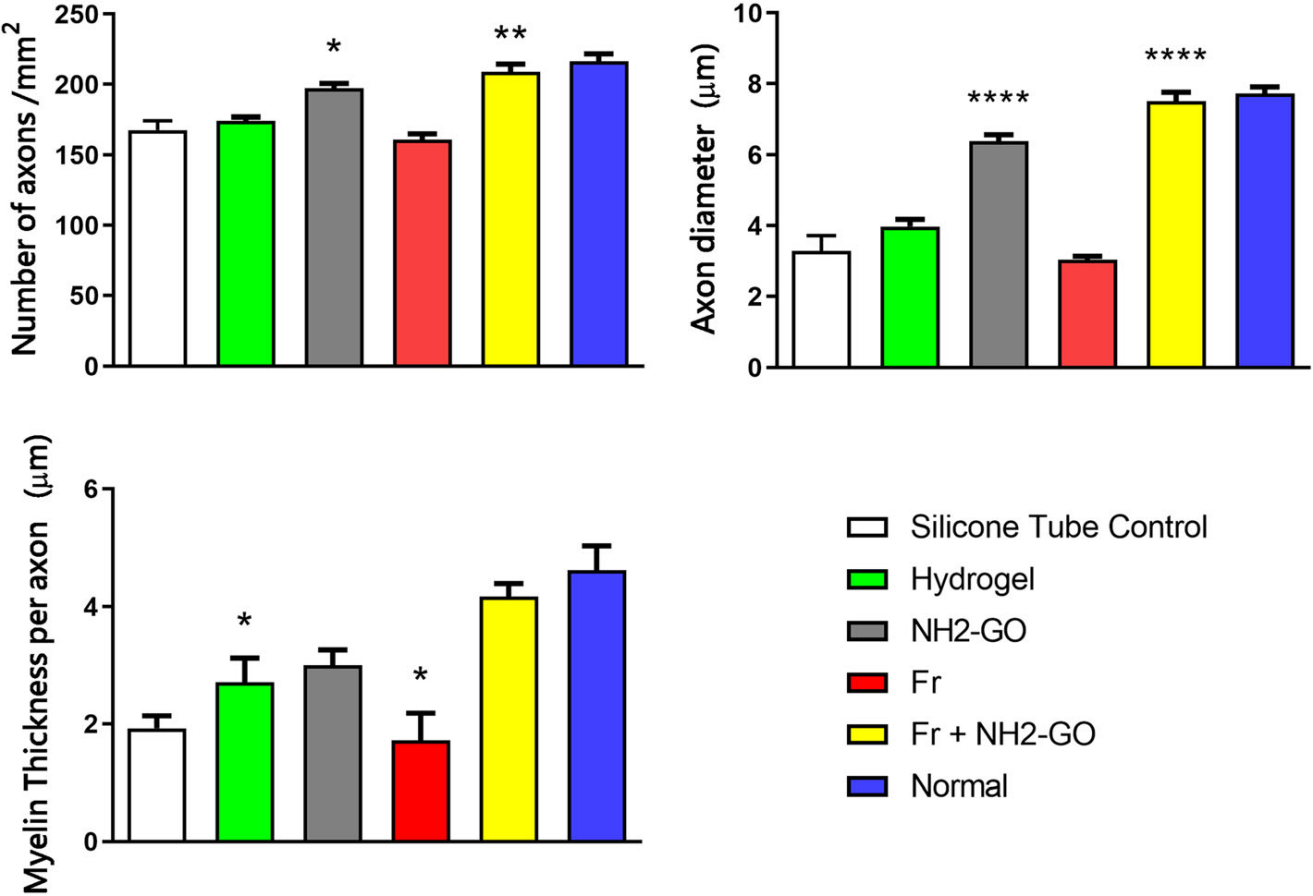
Quantification of nerve regeneration distal to the axotomy site. Axon count, mean diameter, and myelin thickness parameters for all groups were analyzed with one-way ANOVA with Holm-Sidak multiple comparison test used for *p* value output. The data are presented as mean ± standard error of the mean (SEM). *p* values reported above for interventional groups were based on comparison with the hydrogel control group. The results of hydrogel group itself were compared to those of silicone tube control group (*=0.005<*p* value<0.05, **= 0.0005<*p* value<0.005, ***= 0.0001<*p* value<0.0005, ****=*p* value<0.0001). Increased axon count was noticeable both in NH2-GO (*p* value= 0.0252) and Fr + NH2-GO (*p* value=0.0008) groups. Axon diameter was also significantly increased in these groups and reaching similar levels to normal in Fr + NH2-GO group. Moreover, the use of hydrogel improved myelin regeneration (*p* value=0.0189) while graphene oxide failed to further increase this effect. Fr did not prove effective in promoting nerve regeneration and was detrimental to myelin synthesis

**Fig. 5:**
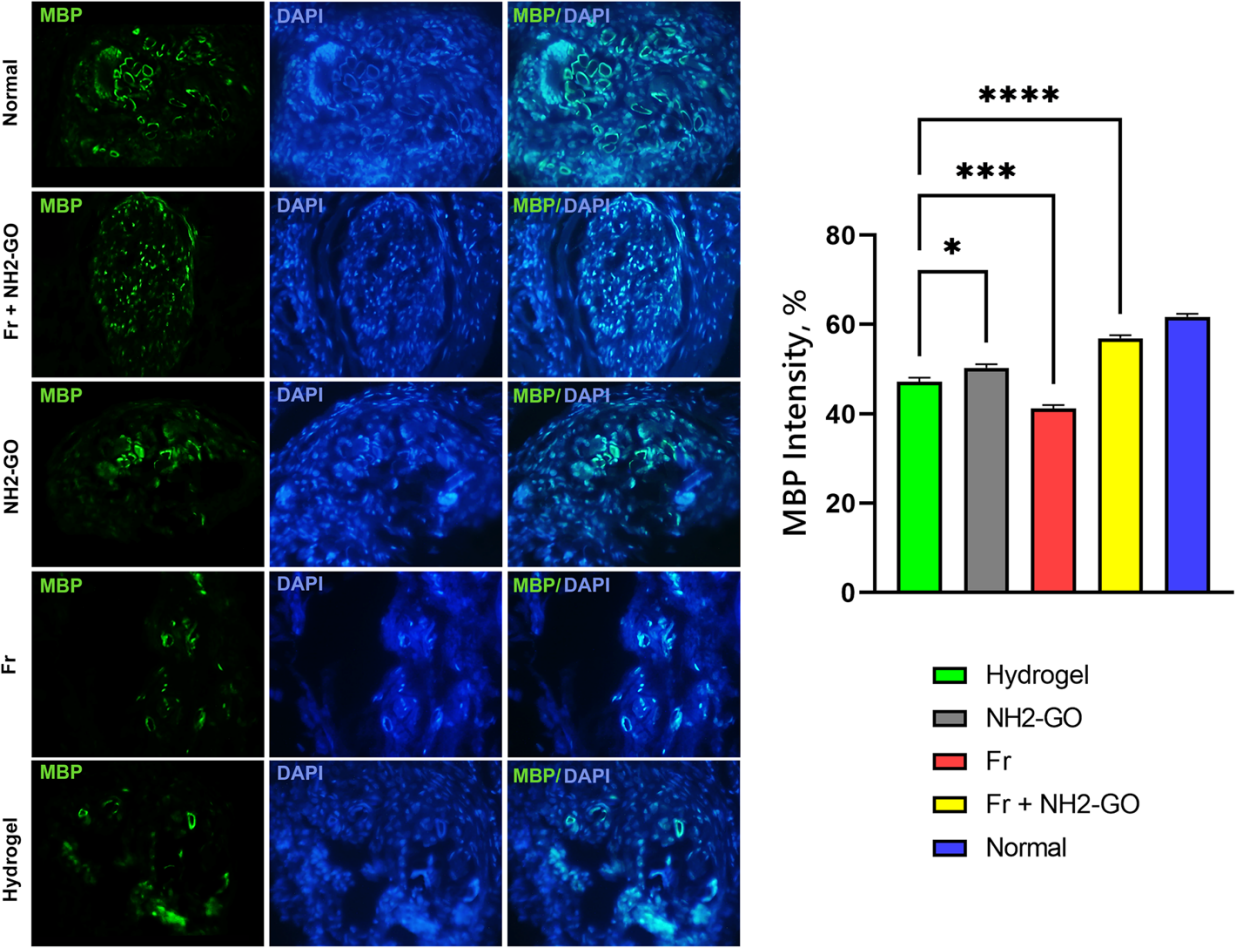
Immunohistochemical staining of horizontal nerve sections distal to the silicone tube conduit with MBP/DAPI. There was a significant increase in myelination in NH2-GO and Fr + NH2-GO marked by increased intensity of the fluorescent marker. The intensity of MBP positive area in the photomicrographs was analyzed using one-way ANOVA and Tukey test multiple comparison. (*=0.005<*p* value<0.05, **= 0.0005<*p* value<0.005, ***= 0.0001<*p* value<0.0005, ****=*p* value<0.0001)

Examination of the LFB-stained horizontal sections of nerve tissue (Fig. 3) showed decreased remyelination of axons in the Fr group compared to hydrogel group, comparable to those of silicone tube control group. The hydrogel control group and other experimental groups, however, showed increased regeneration compared to silicone tube controls. The increased effect of hydrogel adjuncts on tissue regeneration was only observed in NH2-GO group, characterized by increased number of neurite regrowth. The combined use of Fr and NH2-GO dramatically increased both myelination and nerve regrowth compared to hydrogel, NH2-GO, and Fr groups (Fig. 4). The immunohistochemical analysis of regenerated nerves revealed significantly higher intensity of MBP marker in NH2-GO (*p* value=0.013) and Fr + NH2-GO (*p* value <0.0001) groups but lower intensity in Fr group (*p* value=0.0002) compared to hydrogel group (Fig. 5).

**Fig. 6:**
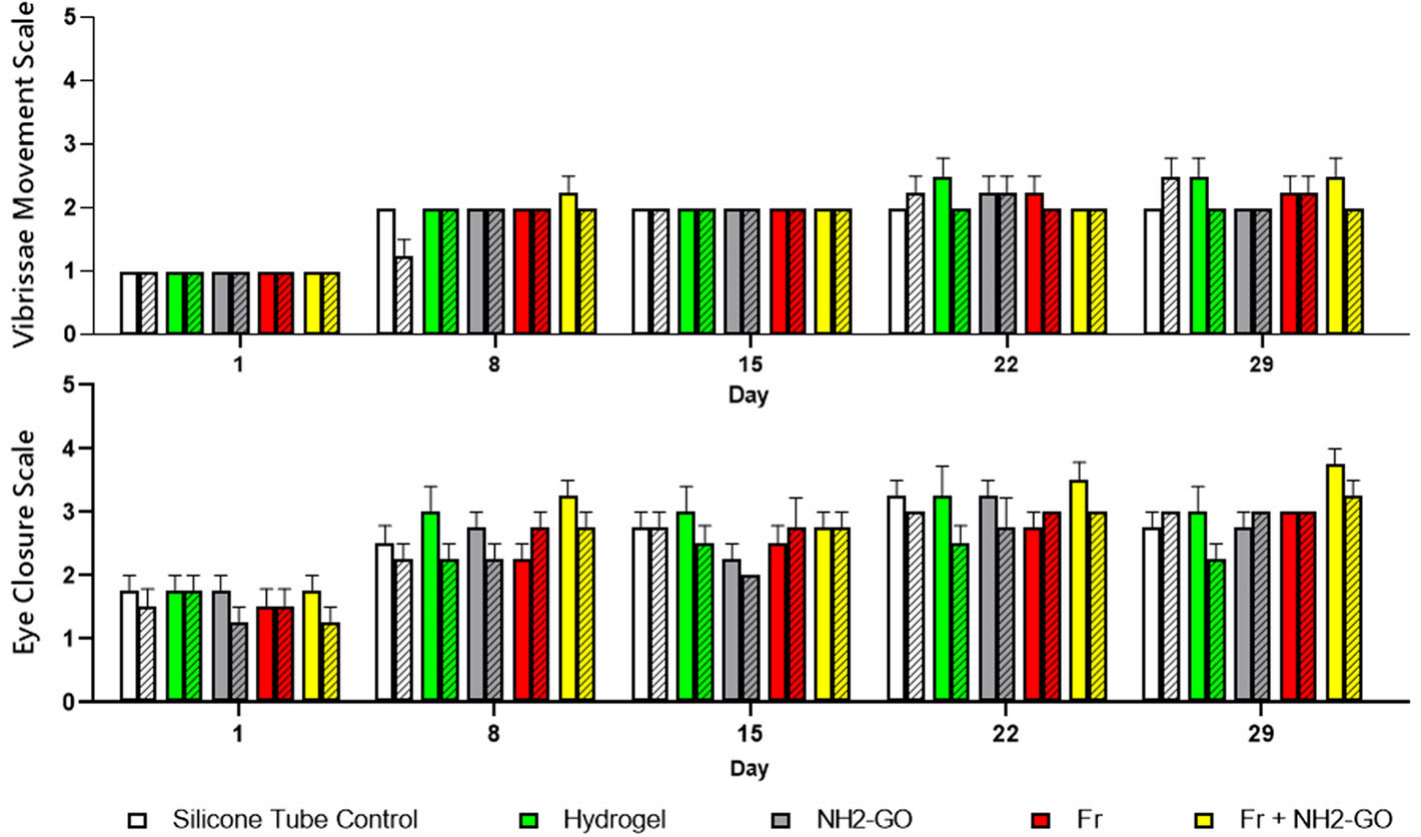
Comparative graph of behavioral test results. Clear and shaded bars represent the scores of the left and the right side of the face, respectively. Error bars represent SEM. The data were analyzed with two-way ANOVA with Tukey multiple comparison test used for *p* value output. No statistically significant changes were reported between groups

The analysis of behavioral test results failed to report any significant improvement between hydrogel and silicone tube control groups and the intervention groups (two-way-ANOVA group factor *p* values 0.38 and 0.39 for eye closure and vibrissae movement tests, respectively). The results were also statistically identical for each group when compared to the contralateral side of the face. Nevertheless, in the final assessment of eye closure and blinking reflex, the left side of face of rats in Fr + NH2-GO group scored the highest vs. other groups (Fig. 6).

## Discussion

Even though peripheral nerves show some regenerative capacities, the success of the nerve repair relies on the surgical approach following an extensive injury. Whereas transposition and nerve grafting each have their advantages, they involve the loss of functionality in less significant muscles such as the muscles of the tongue [46] and masseter [47] when the 12th cranial nerve and the 5th cranial nerve are utilized, respectively. These surgical modalities are also limited by the extent of damage after severe loss of substance and the availability of autologous nerve grafts [6]. Although silicone tubes as a non-degradable synthetic nerve guide require a second surgery to be removed [8], they can make up for their disadvantage with the ease of access and lower financial burden compared to degradable synthetic conduits. When tubulization is coupled with the introduction of extracellular matrix components and neuroregenerative substances into the conduit, it can significantly improve the outcome of nerve repair by providing cell attachment sites for the axons to regrow [48, 49]. Our histological results demonstrated a significant increase in myelination of axons when the chitosan-collagen hydrogel was added inside the silicone tube. However, the rise in number of nerve fibers and their diameter vs. the silicone tube control group did not reach statistical significance. Interestingly, previous studies have reported increased myelinated axonal count when tubulization was carried out with a collagen-laminin-fibronectin-based hydrogel in a rat sciatic nerve axotomy model across a larger gap [50]. In vitro studies have also reported increased neurite outgrowth with collagen gels and higher neuronal growth with chitosan [49, 51].

As previously reported, incorporation of GO in scaffolds improved axonal regeneration after nerve injury [10, 21]. While NH2-GO used in this study did not improve the myelination of regenerated axons in light microscopy, the overall myelination in each biopsy was significantly higher based on the data derived from the immunostaining analysis. We initially hypothesized that bioactive components in Fr extract would potentiate neurite regrowth and myelination by mitigating the axotomy-induced inflammation and promoting Schwann cell proliferation; but intriguingly, Fr extract significantly reduced remyelination and provided no benefit to nerve regeneration. While it can be surmised that the immune reaction to undesired components of the extract outweighed the anti-inflammatory properties of boswellic acids, this was not observed later, where Fr extract was paired with NH2-GO. This observation could be the result of unfavorable byproducts of natural extracts, but the results of this study does not support this hypothesis. Nevertheless, co-administration of these substances synergistically improved both remyelination and axonal regrowth. The precise mechanism of this synergism is not understood but could be postulated to be a result of KBA-mediated increase in NRF2-regulated anti-oxidants in the setting of oxidative stress induced by GO and nerve damage.

The inconsistencies between the results of behavioral and histological tests in this study could be due to a number of possible causes, including low animal count in each group, the nature of the reinnervation process, and potential facial nerve-independent innervation of eyelid closure [52, 53]. Buccal and marginal nerve branches diverged from the facial nerve main trunk converge again to form the distal pes and innervate the whisker pad in rats [44]. The projected axons from the proximal part of the nerve following injury may branch off and inappropriately innervate facial muscles, leading to synkinesis [52]. The regenerated axons may also have trouble re-establishing connections with muscles [54]. Therefore, this aberrant regeneration of nerve fibers leads to inconclusive results in the assessment of reinnervation of facial muscles in animal models. Evidence also exists of a retractor bulbi muscle-mediated eye closure in rats [53]. The incomplete loss of blink reflex in the initial days following the axotomy in our study corroborates this possibility. Future studies should consider the possible interference by these mechanisms and utilize tests with higher sensitivity.

## Conclusions

Our study has shown GO and Fr extract to have significant neuroregenerative properties when used together as an adjunct to hydrogels in a Wistar rat motor neuron axotomy model. Further studies are needed to uncover the synergistic mechanisms and their therapeutic potential in clinical applications; especially in spinal cord injuries, where nerve regeneration is normally restricted.

## Data Availability

The datasets used and/or analyzed during the current study are available from the corresponding author on reasonable request.

## Abbreviations

GO: Graphene oxide
ERK, MAPK: Mitogen-activated protein kinase
ROS: Reactive oxygen species
NH2-GO: Ammonia-functionalized GO
Fr: Frankincense
AKBA: 3-Acetyl-11-keto-β-boswellic acid
KBA: 11-Keto-β-boswellic acid
NRF2: Nuclear factor erythroid-2-related factor 2
MBP: Myelin basic protein
